# Increasing the Duration of Light Physical Activity Ameliorates Insulin Resistance Syndrome in Metabolically Healthy Obese Adults

**DOI:** 10.3390/cells9051189

**Published:** 2020-05-11

**Authors:** Fatema Al-Rashed, Abdulwahab Alghaith, Rafaat Azim, Dawood AlMekhled, Reeby Thomas, Sardar Sindhu, Rasheed Ahmad

**Affiliations:** 1 Immunology & Microbiology Department, Dasman Diabetes Institute, Kuwait 15462, Kuwait; rafaatazim27@gmail.com (R.A.); dalmekhled@gmail.com (D.A.); Reeby.Thomas@dasmaninstitute.org (R.T.); 2 Kuwait Ministry of Health, Mubarak Al-Kabeer Hospital, Immunology Department, Kuwait 13001, Kuwait; 3 Royal College of Surgeons in Ireland (RCSI), D02 YN77 Dublin, Ireland; wahabalghaith@gmail.com; 4 Animal and Imaging Core Facility, Dasman Diabetes Institute, Kuwait 15462, Kuwait; sardar.sindhu@dasmaninstitute.org

**Keywords:** obesity, physical activity, IRS, MHO

## Abstract

Obesity is a well-known risk factor for insulin resistance syndrome (IRS). Nevertheless, limited data are available regarding the effects of physical activity (PA) intensity on the ability to modulate IRS. The study aim was to investigate the beneficial effects of the longer duration of light PA vs. a single bout of the acute moderate or vigorous PA for improvement in IRS indicators. Sixty metabolically healthy obese (MHO) participants, 30 males and 30 females, with body mass index (BMI) of ≥30 were enrolled in this study. PA levels were measured using an accelerometer, and the expression of monocytic surface markers was analyzed using flow cytometry. Plasma cytokines’ secretion was determined by enzyme-linked immunosorbent assay (ELISA). Univariate regression analysis evaluated the actigraphy-assessed PA measures, inflammatory cytokines, and insulin resistance. The longer duration of PA was found to be associated with the homeostatic model assessment of insulin resistance (HOMA-IR), a lower lipid profile, and the expression of inflammatory cytokines by monocytes. Even though, higher intensities of PA were found to be associated with lower body fat percentage, only the light intensity PA was found to be beneficial as it associated with the improved insulin sensitivity and lower expression of inflammatory markers. In conclusion, maintaining the longer duration of low-intensity PA throughout the day could be more beneficial for reducing inflammation and improving insulin resistance. This study supports a more feasible approach model to gain beneficial lifestyle changes for the prevention of IRS in metabolically healthy adults with obesity.

## 1. Introduction

The modern-day obesity pandemic is propagated by increased urbanization combined with unbalanced diets and sedentary lifestyle has resulted in the prevalence of noncommunicable diseases (NCDs) worldwide [1]. While, obesity definition of the body mass index (BMI) as > 30 may seem as an oversimplified definition, it is accurate in capturing and computing the increased risks associated with being overweight or obese and developing NCDs such as type 2 diabetes mellitus (T2DM), dyslipidemia, hypertension, cardiovascular disease (CVD), and sleep apnea [2,3]. In Kuwait, more than 75% of the adult population is currently either overweight or obese [4]; whereas, the prevalence of obesity among children and adolescents under 18 years of age is escalating. NCDs that included cancers, upper respiratory tract diseases, heart disease, and diabetes were estimated to account for 73% of the total deaths in Kuwait in 2013 [5]. Regarding prevalence rates of NCDs risk factors among Kuwaiti population, 63% account for inadequate physical activity (PA), with obesity rates exceeding 44% among women [6].

Adipose tissue is dichotomously characterized as subcutaneous adipose tissue (scAT) and visceral adipose tissue (VAT). The VAT is invariably implicated in the pathophysiology of metabolic disorders such as T2DM and CVD [7]. Interestingly, adipocytes secrete unique cytokines called adipocytokines or adipokines which are essential for energy homeostasis. In obesity, adipokines are dysregulated, whereby inflammatory cytokines such as TNF-α, IL-6, IFN-γ, and IL-1β are upregulated, resulting in a state of chronic low-grade inflammation called metabolic inflammation which underlies pathophysiology of obesity and metabolic syndrome [8,9].

In obesity, the upregulation of proinflammatory and the downregulation of anti-inflammatory adipokines are associated with adipocyte hyperplasia and hypertrophy as well as with macrophage polarization from “alternatively activated” anti-inflammatory M2 to “classically activated” proinflammatory M1 phenotypes which are usually lipid-laden in the adipose tissue from obese and overweight individuals [10,11].

The current framework for clinical obesity management encompasses a wide range of pharmacological, lifestyle, behavioral, and surgical techniques in order to reduce intra-abdominal fat and increase lean mass. Exercise represents an effective lifestyle or non-pharmacological intervention with beneficial immune modulatory effects, including reduced levels of pro-inflammatory and increased levels of anti-inflammatory cytokines [12,13]. However, the effects of exercise (acute single bout) on the immune response differ dramatically from those of training (maintaining exercise for long-term and longer durations). Acute exercise bouts are known to elicit transient increases in proinflammatory cytokines; with elevated levels induced by higher intensity exercises for longer time periods [14,15,16]. It implies that the repeated exposure such as a regular low-intensity exercise training or activity induces an adaptive immune response to attenuate proinflammatory cytokine expression and benefit the host. We hypothesize that the increases in light PA duration mimic the regular training exercise and, therefore, could improve insulin resistance syndrome (IRS) in adult obese population. The current recommendations for the PA of adults aged between 18 and 64 stipulate that a minimum 150 min of moderate PA or 75 min of moderate to vigorous PA per week should be required [17], yet, no specific recommendations have been established for metabolically healthy obese (MHO) adults.

The aim of the study was to investigate the beneficial effects of the longer duration of light PA vs. a single bout of the acute moderate or vigorous PA for improvement in IRS indicators. This study reports objectively measured PA intensity levels in 60 MHO individuals categorized as light, moderate, and high intensity PA, based on Freedson’s cut-offs [18]. Obesity is a potential risk factor for T2DM, CVD, and other IRS disorders. The MHO phenotype has been identified and defined as the presence of obesity in the absence of metabolic dysregulation i.e., no cardiometabolic risk factors exceeding the identified thresholds of systolic and diastolic blood pressure, blood glucose, triglycerides, and high-density lipoprotein cholesterol [19,20]. Therefore, it becomes important to characterize the immuno-metabolic differences between healthy and non-healthy obese populations. In addressing our core question regarding how different PA intensity levels can impact health indicators in the MHO subjects, we herein investigate the associations between PA intensities and immuno-metabolic markers that are known to be related with IRS.

## 2. Materials and Methods

### 2.1. Study Design, Participants, and Anthropometric Measurements

This was a cross-sectional observational study of non-smoking, non-drinking, MHO adults with no past or current medical disorders, and with a BMI equal to or greater than 30 (kg/m^2^). All participants were informed about the study and the risks involved, before signing a written consent form and doing a health screen. A total of 60 participants (30 male and 30 female) with a mean age of 33.2 ± 3.49 years were recruited. The study was approved by the Kuwait Ministry of Health (MOH) Ethics Board (2017/542). None of the participants had physical disabilities that would prevent or severely limit physical mobility or PA. A standard protocol for anthropometric assessments of all subjects was used, using the same equipment throughout the study. In brief, height and weight measurements were conducted in private settings. After measuring physical activity for seven consecutive days, participants were asked to visit the lab early in the morning dressed in light clothing. After resting for 10 min, three consecutive blood pressure measurements were taken (with 1 min interval). Height was measured to the nearest 1.0 cm using portable stadiometer. Weight was measured to the nearest 0.2 kg with standard physician’s beam scale (Detecto). Body mass index (BMI) was calculated as body weight in kilograms divided by height in meters squared (kg/m^2^). Participants were then taken to the phlebotomy unit for collection of blood samples.

### 2.2. PA Measurements

All participants were given an electronic accelerometer (Actigraph GT3X; Actigraph LLC, Pensacola, FL, USA). Subjects were advised to maintain their normal daily (habitual) PA levels during the study period. Accelerometers attached to elasticized belts were worn on right-side hips for seven consecutive days (except when bathing and during water activity). The actigraphy method enabled for a reliable and objective assessment of the daily PA [21,22]. The accelerometer provided PA measurements that included activity counts, vector magnitude, energy expenditure, step counts, activity intensity levels, and metabolic equivalents (METs). A 1-min epoch was used in this study, with activity counts assessed at 1-min intervals to ensure that the data quality for the participants included at least four days during which the accelerometer was worn for at least 60% of the day. A non-wear time was taken as any block of time ≥60 min where the activity count was equal to zero.

Freedson’s cut-offs [18] were used to differentiate between the PA intensity levels including light-intensity activity (100–1951 counts/min), moderate-intensity activity (1952–5724 counts/min), and vigorous-intensity activity (≥5725 counts/min). All counts lower or equal to 99 count/min were considered as a sedentary status. The data were also expressed as mean intensity of each activity during the monitoring time i.e., total accelerometer counts per total monitoring time.

### 2.3. Measurement of Blood Metabolic Markers

Participants were asked for the visit after an overnight fasting for at least 10 h. Three consecutive blood pressure measurements, along with heart rate, were taken for each individual. Blood samples were collected in 10 mL EDTA tubes (BD Vacutainer system, Plymouth, UK). Plasma was separated and frozen immediately at −80 °C for further analysis.

Blood glucose, fasting insulin, cholesterol, high-density lipoprotein (HDL)-cholesterol, and triglycerides were determined by biochemical analysis using a single assay upon the completion of the study. Quality control sera were used to monitor the accuracy and precision of the assays performed. Homeostatic model assessment of insulin resistance (HOMA-IR) index was used as a measure of insulin resistance and calculated from the basal (fasting) glucose and insulin concentrations using the following formula:

HOMA-IR = fasting insulin (μU/L) × fasting glucose (nmol/L)/22.5

### 2.4. Flow Cytometry for Immune Cell Markers

To determine monocyte/macrophage lineages in the whole blood, multicolor fluorescence-activated cell sorting (FACS) analysis was conducted using freshly collected whole blood samples. Briefly, 1 mL of lysing buffer was added to 0.1 mL of blood sample to eliminate erythrocytes and the remaining peripheral blood mononuclear cells (PBMCs) populations were incubated with fluorochrome-conjugated mouse anti-human monoclonal antibodies against CD14, CD16, CD163, CD206, CD11c, and HLA-DR as well as with isotype-specific antibodies for respective controls (BD Pharmingen, San Diego, CA, USA). The three gating strategies used in this study included (i) evaluation of CD14^+^ cells within the total leukocyte population and in CD14+ cell population, circulating non-classical or intermediate monocytes subsets were further identified by expression of CD16; (ii) identifying M2 cell populations within the CD14^+^ cells by their expression of CD163 and CD206; and (iii) identifying M1 cell populations within the CD14^+^ monocytes/macrophages by expression of CD11c and HLA-DR surface markers. Data were collected using a BD FACSCanto II flow cytometer and analyzed using DIVA software (version V6.1.3, BD Pharmingen).

### 2.5. Enzyme-Linked Immunosorbent Assay (ELISA) for Measuring Soluble Proteins and Inflammatory Cytokines/Chemokines

Commercially available enzyme-linked immunosorbent assay (ELISA) kits were used for detection of plasma insulin, C-peptide levels (Mercodia, Uppsala, Sweden) as well as circulatory levels of inflammatory cytokines/chemokines or factors including IL-1β, TNF-α, IL-17A, MCP-1, and VEGF (R&D Systems, Minneapolis, MN, USA), following instructions from the manufacturers.

### 2.6. Statistical Analysis

The data obtained were expressed as mean ± SD values. For statistical analysis, GraphPad Prism (version 6.05; San Diego, CA, USA) and SPSS for Windows version 19.01 (IBM SPSS Inc., Armonk, NY, USA) software were used. Pearson’s correlation coefficient ‘r’ was used to assess the linear association and Multiple linear regression analysis was adjusted for the potential confounders to examine associations between monocyte surface marker expression and metabolic risk variables that were found to be associated with higher durations of PA. All *p*-values < 0.05 were considered statistically significant and expressed as follows: NS: not significant, *p < 0.05, ** *p* < 0.01, *** *p* < 0.001, and **** *p* < 0.0001)

## 3. Results

### 3.1. Demographics and Clinical Characteristics of the Study Population

Descriptive statistics of the study participants are summarized in Supplementary Table S1. Since no significant differences were found between both gender subgroups regarding BMI and lipid profiles, subsequent analyses data of male and female were pooled. Objectively evaluating the levels and intensities of PA using Freedson’s cut-offs [18], the participants spent, on average, most of their day either being sedentary (including sleeping time) (70.6% ± 4.1%) or conducting light activities (22.9% ± 3.5%). Only 5.3% ± 1.4% of wearing time per day was spent conducting moderate intensity PA, and even lesser time was used for vigorous-intensity PA (0.91% ± 0.71%) which is equivalent to 1.5 h.

To understand how different trends of PA were associated with metabolic variables and inflammatory responses, Pearson’s bivariate correlation was determined among three different levels of PA intensity, that is, light intensity PA (LPA), moderate intensity PA (MPA), and high intensity PA (HPA) (Table 1). A negative association was found for body fat weight (r = −0.026, *p* = 0.04), and fat percentage (r = −0.32, *p* = 0.01) with longer duration of moderate-intensity PA. No significant association was found between total duration or intensity of PA and BMI.

We then investigated the effects of PA trends and how they were associated with known continuous metabolic risk variables (Table 2). A significant negative association (r = −0.53, *p* ≤ 0.05) was observed between increased duration of light/moderate intensity PA (LPA and MPA) and cardiac health (blood pressure and heart rate). Nonetheless, when we looked at the association between PA intensity and lipid profiles, we found that only the LPA seemed to be associated with lower triglyceride levels (r = 0.37, *p* = 0.02). Interestingly, LPA was found to be strongly associated with increased insulin sensitivity as indicated by HOMA-IR (r = −0.37, *p* = 0.005) and circulatory C-peptide levels (r = −0.31, *p* = 0.04), with no associated effects observed regarding MPA and HPA.

### 3.2. Monocyte Subset Markers and Plasma Cytokines

CD14 and CD16 surface expression was measured by flow cytometry to identify the circulating monocyte subsets as (i) classical monocytes (CD14^++^CD16^−^); (ii) intermediate monocytes (CD14^++^CD16^+^) and (iii) non-classical monocytes (CD14^dim^CD16^++^) (Supplementary Figure S1). The data show that non-classical monocyte expression was strongly associated with total duration of PA (r = −0.53, *p* = 0.007), and a longer duration of LPA (r = −0.48, *p* = 0.01). Further analysis of the M1 (CD14^+^CD11c^+^HLA-DR^+^) and M2 (CD14^+^CD163^+^CD206^+^) phenotypes revealed that all intensity levels of PA had a strong positive association with the increased expression of M2 anti-inflammatory monocytic markers. Conversely, none of the PA levels were associated with the M1 pro-inflammatory monocytic markers (Table 3).

To further study the relationship between intensity levels of PA and inflammatory cytokines/chemokines, we quantified the circulatory levels of signature inflammatory cytokines that are known to be typically involved in obesity-related complications (Table 4). A negative association was found between total duration of PA (regardless of the intensity level) and plasma TNF-α levels. Both IL-17A and MCP-1 levels were found to be negatively associated with total duration of PA (r = −0.4, *p* = 0.02 and r = −0.42, *p* = 0.02, respectively); as well as with higher duration of LPA (r = −0.38, *p* = 0.037 and r = −0.48, *p* = 0.007, respectively).

We next wanted to investigate the independent associations between expression of monocyte surface markers (CD14^dim^CD16^++^/CD14^+^CD206^+^CD163^+^) and metabolic parameters or inflammatory status within the LPA group. Multiple regression analysis showed that only the expression of CD14^dim^CD16^++^ monocyte markers associated independently with resting heart rate, HOMA-IR, and C-peptide; while, no associations were found regarding M2 markers expression (Table 5).

### 3.3. Association between CD14^dim^CD16^++^ Monocyte Markers Expression and Various IRS Markers

**Figure 1:**
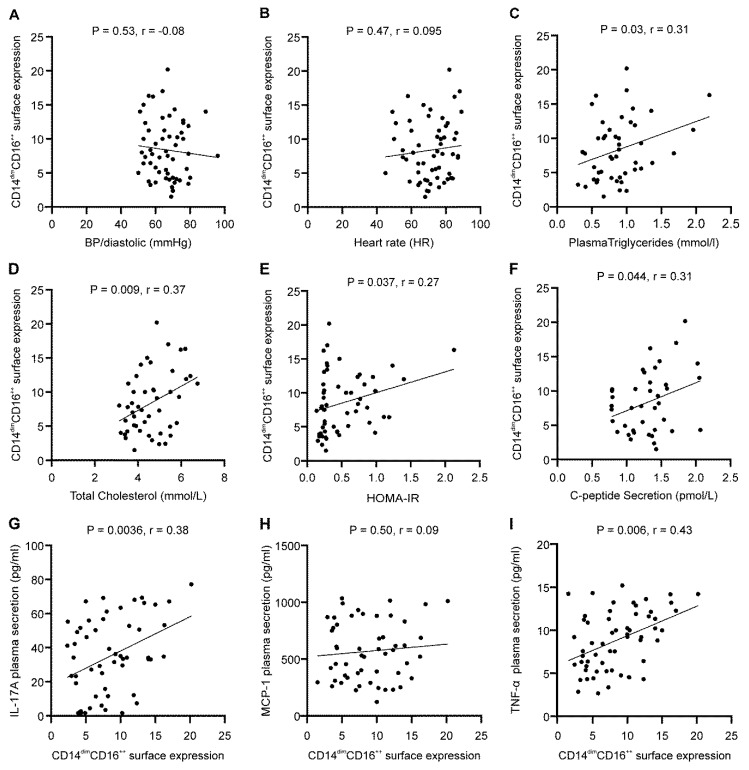
(**A**–**I**) Association between CD14^dim^CD16^++^ monocyte subsets and insulin resistance syndrome (IRS) markers. Multiple linear regression analysis between non-classical blood monocyte counts and the changes in metabolic and inflammatory markers within the longer duration of PA group. All data are expressed as mean ± SD. *p* ≤ 0.05 was considered statistically significant (* *p* < 0.01; ** *p* < 0.001, *** *p* < 0.0001).

The CD14^dim^CD16^++^ monocyte counts were increased with longer durations of total PA and LPA. To understand the clinical significance of this change, we conducted the Pearson’s bivariate correlation analysis and found that the non-classical blood monocyte counts and changes in metabolic and inflammatory markers associated with longer duration of PA (Figure 1). No association was found between non-classical blood monocyte counts and cardiac health (assessed by diastolic blood pressure or heart rate measurements) (Figure 1A,B). In contrast, a positive association of CD14^dim^CD16^++^ blood monocyte counts were found with triglycerides (r = 0.31, *p* = 0.03) and total cholesterol (r = 0.37, *p* = 0.009) (Figure 1C,D). A positive association was also observed between non-classical blood monocyte counts and glucose tolerance measured as HOMA-IR (r = 0.27, *p* = 0.037) and C-peptide secretion (r = 0.31, *p* = 0.04) (Figure 1E,F). Furthermore, only IL-17A (r = 0.38, *p* = 0.003) and TNF-α (r = 0.43, *p* = 0.006) correlated positively with the CD14^dim^CD16^++^ blood monocyte counts; no association was found with the MCP-1 expression (r = 0.09, *p* = 0.50) (Figure 1G–I).

## 4. Discussion

Our data show that the increasing amounts of light to moderate PA intensities are more beneficial for cardiovascular health in otherwise non-active obese individuals. We also found a negative association between lower diastolic BP (mmHg) and lower heart rate (HR), corresponding to the longer duration of LPA. Consistent with our findings, at least in part, several previous studies have shown that moderate intensity PA was protective of cardiometabolic risk factors in obese individuals [23,24,25]. In line with this, the Canadian society for exercise physiology recommends a comprehensive approach to quantify movements that involve sleep, sedentary time, moderate-to-vigorous PA, light daily activities [26,27].

Obesity is associated with increase in BMI, elevation of inflammatory responses [28], and adipocyte enlargement, which have been associated with changes in monocyte/macrophage polarization [29]. In regard to BMI, in our study participants, no significant association was found between the total duration of PA (at all intensities) and the BMI. Notably, a significant association was found between the longer duration of moderate-intensity exercise and the body fat level which indicated that the lighter activities, regardless of their duration, might not induce significant benefits with respect to body weight or fat levels. These findings were similar to that of other epidemiological prospective studies and the National Consensus guidelines that recommended extended time periods of moderate-to-high intensity workout to maintain body weight and a healthy lifestyle [17,30,31].

A negative association was found between light-to-moderate PA intensity levels and total cholesterol, though statistical significance was not reached (r = 0.3, *p* = 0.06 and r = −0.31, *p* = 0.059). A dose–response inverse relationship between PA and cardiovascular health has been reported [32,33]; while on the other hand, an association was also found between vigorous PA and a reduced risk of heart failure [34,35]. This discrepancy could be related to a plausible difference in the mechanisms via which PA could modify the cardiovascular risk factors, such as blood pressure and lipid profile [36], or via noncardiac mechanisms including the lungs, skeletal muscles, and glucose homeostasis [37,38]. Importantly, HOMA-IR and circulatory C-peptide levels reveal strong associations among longer duration of PA, glucose tolerance, and insulin sensitivity. However, the current study found that only the increased duration of light intensity PA was associated with lower HOMA-IR and C-peptide levels. A previous study showed that increased C-peptide levels related with the hazards of cardiovascular and death in non-diabetic adults and were a better predictor of these outcomes than serum insulin and/or glucose derived indicators [39].

The effect of PA intensity on glucose tolerance and sensitivity has long been debated. Herzig et al. [40] showed that high-risk participants who engaged in 3 months of light-intensity walking exercise (equivalent to ≈3 km h^−1^) had significantly decreased fasting glucose, HOMA-IR, total cholesterol, and visceral fat. Conversely, Cockcroft et al. debated that a single bout of high-intensity activity was more beneficial in improving glucose tolerance compared to a moderate-intensity workout when measured immediately after exercise [41]. Despite these conflicting results, the majority of the research studies are in agreement that the total duration of PA appears to be more important for insulin response than the level of the workout intensity [40,42,43,44].

In the present study, no significant changes were found in M1 pro-inflammatory surface markers expression across different intensities levels of PA. However, a strong positive association was found between total duration of PA at all intensities and M2 anti-inflammatory surface markers expression. Mechanistic studies in humans suggested that PA induced an elevation in the anti-inflammatory/pleiotropic cytokines, such as IL-6 and IL-10, and resulted in the inhibition of TNF-α, which in turn blocked IL-1β signaling by stimulating IL-1ra [45,46,47]. In agreement with these reports, the current study also supports that PA could potentially be used as a natural strategy to improve anti-inflammatory immune responses.

Although an association between PA and M1 monocyte surface markers expression was not observed, we found that longer duration of light intensity PA correlated inversely with the CD14^dim^CD16^++^ monocytic counts in our study population. In healthy humans, circulatory monocyte subsets are characterized as classical monocytes (CD14^+^CD16^-^), intermediate monocytes (CD14^+^CD16^+^), and non-classical monocytes (CD14^dim^CD16^++^); while, each subpopulation can differ in both phenotype and function [48]. Among the three monocyte subsets, the CD16^+^ cells are considered the primary potent inducers of inflammation and are known to be upregulated in obese subjects with insulin resistance [49,50]. Furthermore, we observed that both the total duration and increased duration of light PA were negatively associated with the plasma levels of inflammatory cytokines/chemokines including IL-17A and MCP-1 which are known to be elevated in obesity [51,52].

These results suggested that the association between monocyte subset expression and IRS markers was negatively associated with an extended duration of light PA. We, therefore, conducted an association analysis between the monocyte subset counts and the associated metabolic risk markers. While no direct association was found between the CD14^dim^CD16^++^ monocyte subsets and cardiac health, a significant positive association was found with the lipid profile, such as glucose tolerance, and some inflammatory cytokines (IL-17A and TNF-α). These data could possibly explain why some variation was reported regarding the PA and impact on cardiac health. It was previously reported that physically active individuals possessed lower numbers of non-classical CD16^+^ monocytes and a single exercise session modulated function and improved total counts of those subsets [53,54]. Another study reported that the insulin-resistant and not the insulin-sensitive obese individuals had increased percentages of CD16^+^ monocytes which could be slightly modulated by a single bout of moderate aerobic exercise [55]. In our study, however, we show that in MHO individuals, increasing the duration of PA in the milder intensity was enough to modulate the CD16^+^ monocyte subset. We speculate that these findings may be clinically relevant to the population studied since this effect on CD16^+^ monocyte subset could contribute, directly and/or indirectly, to the maintenance of an MHO phenotype and insulin sensitivity.

Nonetheless, our study is limited by a few caveats. First, in this study, we included only the non-smokers and non-drinkers with no past or current medical complications and these stringent requirements obviously reduced the total number of participants enrolled, thus resulting in a small study population. Second, we did not include the waist to hip ratios (i.e., central obesity) in our analysis, which meant that we were unable to analyze the similar associations regarding IRS markers and inflammation in the context of abdominal obesity. Third, this study was a cross sectional study by design and, therefore, long-term follow-up studies involving (i) a larger numbers of participants; (ii) lean/overweight healthy individuals; and (iii) metabolically unhealthy obese (MUO) cohort will be required in the future in order to generate more robust and comprehensive data across a longer timeline.

## 5. Conclusions

The present study shows that a longer duration of PA per day was associated with the improved cardiovascular health, favorable lipid profile, glucose tolerance, and insulin sensitivity. Interestingly, our data show that increasing amounts of light intensity PA were more beneficial for cardiovascular and immuno-metabolic health in otherwise non-active obese individuals. The data support that MHO population benefits more from the extended periods of light PA compared to short bouts of moderate-to-high intensity exercise. These findings support a novel approach for the prevention of obesity-related complications in metabolically healthy obese (pre-diabetic) individuals, implying that maintaining the light-intensity daily PA could replace a vigorous workout for being a more viable PA intervention, especially for those with heart, foot, or leg problems that may preclude them from doing aerobic exercise.

## Figures and Tables

**Table 1:** Regression analysis of the percentage of time spent at different physical activity (PA) intensity levels and anthropometric/body composition characteristics

	Standardized Regression Coefficients (95% CI)				*p*-Value				
	Mean ± SD	Overall Activity (%)		Light Intensity (%)		Moderate Intensity (%)		High Intensity (%)	
**Age (years)**	33.2 ± 3.4	0.06 (−0.18 to 0.31)	0.59	0.11 (−0.14 to 0.36)	0.37	−0.09 (−0.34 to 0.16)	0.45	0.02 (−0.23 to 0.27)	0.84
**Weight (kg)**	93.2 ± 11.8	−0.02 (−0.28 to 0.22)	0.83	−0.07 (−0.32 to 0.18)	0.58	−0.01 (−0.27 to 0.24)	0.90	−0.04 (−0.29 to 0.21)	0.76
**Height (cm)**	166.4 ± 10.3	0.21 (−0.04 to 0.44)	0.10	0.08 (−0.18 to 0.32	0.54	0.28 (0.03 to 0.50)	0.026 *	0.15 (−0.10 to 0.39	0.23
**BMI (kg/m^2^)**	33.3 ± 2.6	−0.14 (−0.38 to 0.11)	0.26	−0.12 (−0.36 to 0.13)	0.36	−0.17 (−0.41 to 0.08)	0.18	−0.13 (−0.37 to 0.12)	0.31
**Waist circumference (inch)**	41.5 ± 4.9	−0.10 (−0.34 to 0.15)	0.44	−0.09 (−0.34 to 0.16)	0.47	−0.13 (−0.38 to 0.12)	0.30	−0.11 (−0.35 to 0.14)	0.40
**Hip circumference (inch)**	46.6 ± 3.4	−0.08 (−0.32 to 0.17)	0.53	−0.00 (−0.26 to 0.24)	0.96	−0.17 (−0.41 to 0.08)	0.18	−0.20 (−0.43 to 0.05)	0.12
**Fat weight (kg)**	38.6 ± 6.6	−0.32 (−0.53 to −0.08)	0.01 **	−0.20 (−0.44 to 0.05)	0.113	−0.26 (−0.48 to −0.01)	0.04 *	−0.16 (−0.40 to 0.09)	0.20
**Lean weight (kg)**	54.5 ± 9.7	0.18 (−0.07 to 0.41)	0.16	0.10 (−0.15 to 0.35)	0.43	0.23 (−0.01 to 0.46)	0.06	0.10 (−0.15 to 0.35)	0.41
**Fat %age**	36.84± 5.0	−0.29 (−0.51 to −0.04)	0.02 *	−0.19 (−0.42 to 0.06)	0.14	−0.32 (−0.53 to −0.07)	0.01 *	−0.20 (−0.43 to 0.05)	0.11

BMI: body mass index; CI: confidence interval; SD: standard deviation; * *p* < 0.05, ** *p* < 0.01.

**Table 2:** Regression analysis of the percentage of time spent at different intensity levels of PA and metabolic risk variables.

	Standardized Regression Coefficients (95% CI)				*p*-Value				
	Mean ± SD	Overall Activity (%)		Light Intensity (%)		Moderate Intensity (%)		High Intensity (%)	
**BP/systolic (mmHg)**	118.4 ± 9.3	−0.17 (−0.41 to 0.09)	0.2	−0.10 (−0.35 to 0.15)	0.42	−0.21 (−0.44 to 0.04)	0.10	−0.05 (−0.30 to 0.20)	0.70
**BP/diastolic (mmHg)**	73.6 ± 9.3	−0.42 (−0.61 to −0.19)	0.001 ***	−0.32 (−0.53 to −0.07)	0.01 **	−0.40 (−0.59 to −0.15)	0.002 **	−0.17 (−0.41 to 0.08)	0.19
**HR**	76.7 ± 6.7	−0.37 (−0.59 to −0.15)	0.002 **	−0.31 (−0.53 to −0.06)	0.01 **	−0.31 (−0.52 to −0.05)	0.017 *	−0.19 (−0.42 to 0.07)	0.15
**Triglycerides (mmol/L)**	0.97 ± 0.1	−0.35 (−0.60 to −0.03)	0.03 *	−0.37 (−0.62 to −0.05)	0.02 *	−0.13 (−0.43 to 0.20)	0.44	−0.05 (−0.37 to 0.27)	0.74
**Total cholesterol (mmol/L)**	5.2 ± 0.7	−0.38 (−0.63 to −0.07)	0.01 **	−0.30 (−0.57 to 0.023)	0.06	−0.31 (−0.57 to 0.01)	0.059	−0.25 (−0.53 to 0.07)	0.12
**HDL cholesterol (mmol/L)**	1.2 ± 0.2	−0.12 (−0.43 to 0.21)	0.47	-0.01 (−0.34 to 0.31)	0.92	−0.24 (−0.53 to 0.08)	0.14	−0.16 (−0.47 to 0.16)	0.32
**Fasting glucose (mmol/L)**	5.2 ± 0.6	−0.22 (−0.45 to 0.04)	0.10	−0.23 (−0.46 to 0.03)	0.08	− 0.11 (−0.36 to 0.1604)	0.42	0.03 (−0.23 to 0.29)	0.78
**Insulin Con. (mu/)**	2 ± 1.3	0.08 (−0.18 to 0.33)	0.54	−0.05 (−0.31 to 0.21)	0.69	0.24 (−0.02 to 0.47)	0.07	0.30 (0.04 to 0.52)	0.02 **
**HOMA−IR**	0.4 ± 0.3	−0.33 (−0.55 to −0.08)	0.01 **	−0.37 (−0.57 to −0.12)	0.005 **	0.00 (−0.26 to 0.26)	0.98	0.01 (−0.24 to 0.28)	0.89
**C−Peptide (pg/mL)**	1.5 ± 0.4	−0.34 (−0.59 to −0.03)	0.02 *	−0.31 (−0.57 to −0.00)	0.04 *	− 0.10 (−0.39 to 0.21)	0.53	− 0.30 (−0.56 to 0.00)	0.053

BP: blood pressure; HR: heart rate; HDL: high-density lipoprotein; HOMA-IR: homeostatic model assessment of insulin resistance; CI: confidence interval; SD: standard deviation; * *p* < 0.05, ** *p* < 0.01, *** *p* < 0.001.

**Table 3:** Regression analysis of the percentage of time spent at different intensity levels of PA, metabolic risk variables, and monocytic cell surface markers

	Standardized Regression Coefficients (95% CI)				*p*-Value				
	Mean ± SD	Overall Activity (%)		Light Intensity (%)		Moderate Intensity (%)		High Intensity (%)	
**Classical (%)** **(CD14+CD16-)**	64.1 ± 17.6	−0.14 (−0.51 to 0.27)	0.50	−0.12 (−0.50 to 0.29)	0.56	−0.11 (−0.49 to 0.30)	0.59	−0.00 (−0.40 to 0.39)	0.97
**Intermediate (%)** **(CD14+CD16+)**	7.8 ± 2.4	0.02 (−0.38 to 0.41)	0.92	0.08 (−0.32 to 0.47)	0.69	−0.06 (−0.45 to 0.34)	0.75	−0.15 (−0.52 to 0.26)	0.46
**Non-classical (%)** **(CD14dimCD16++)**	13.9 ± 16.9	−0.53 (−0.77 to −0.16)	0.007 **	−0.48 (−0.74 to −0.10)	0.01 **	−0.28 (−0.62 to 0.12)	0.17	−0.34 (−0.65 to 0.07)	0.10
**CD14+CD163+CD206+ (%)** **(M2 subset expression)**	8.1 ± 9.4	0.85 (0.60 to 0.95)	<0.0001 ****	0.69 (0.25 to 0.89)	0.006 **	0.67 (0.22 to 0.88)	0.008 **	0.62 (0.03 to 0.83)	0.04 *
**CD14+CD11c+HLA-DR+ (%)** **(M1 subset expression)**	93.8 ± 3.8	0.19 (−0.28 to 0.59)	0.41	0.33 (−0.14 to 0.68)	0.16	−0.16 (−0.57 to 0.31)	0.51	−0.00 (−0.45 to 0.45)	0.99

CI: confidence interval; SD: standard deviation; *p < 0.05, ** *p* < 0.01, *** *p* < 0.001, and **** *p* < 0.0001).

**Table 4:** Regression analysis of the percentage of time spent at different intensity levels of PA, metabolic risk variables, and inflammatory cytokines/chemokines in the circulation.

	Standardized Regression Coefficients (95% CI)				*p*-Value				
	Mean ± SD	Overall Activity (%)		Light Intensity (%)		Moderate Intensity (%)		High Intensity (%)	
**TNF-α (pg/mL)**	64.1 ± 17.6	−0.53 (−0.74 to −0.21)	0.002 **	−0.44 (−0.69 to −0.09)	0.01 **	−0.37 (−0.65 to −0.02)	0.039 *	−0.40 (−0.66 to −0.04)	0.03 *
**Il−1β (pg/mL)**	7.8 ± 2.4	0.04 (−0.32 to 0.39)	0.82	−0.02 (−0.38 to 0.33)	0.88	0.21 (−0.15 to 0.53)	0.25	0.08 (−0.28 to 0.42)	0.66
**IL−6 (pg/mL)**	13.9 ± 16.9	−0.25 (−0.55 to 0.10)	0.16	−0.31 (−0.60 to 0.04)	0.09	−0.01 (−0.36 to 0.34)	0.95	0.14 (−0.21 to 0.47)	0.43
**IL−17A (pg/mL)**	48.8 ± 18.8	−0.4 (−0.66 to −0.045)	0.03 *	−0.38 (−0.65 to −0.02)	0.04 *	−0.21 (−0.53 to 0.15)	0.24	−0.14 (−0.48 to 0.22)	0.43
**MCP−1 (pg/mL)**	456.1 ± 293.1	−0.42 (−0.67 to −0.07)	0.02 *	−0.48 (−0.71 to −0.14)	0.007 **	−0.01 (−0.36 to 0.35)	0.95	−0.08 (−0.43 to 0.28)	0.65
**VEGF (pg/mL)**	652.4 ± 330.9	0.02 (−0.33 to 0.38)	0.90	0.00 (−0.35 to 0.36)	0.99	0.13 ( −0.24 to 0.46)	0.48	−0.07 (−0.42 to 0.28)	0.68

TNF-α: tumor necrosis factor-alpha; IL: interleukin; MCP-1: macrophage chemotactic protein-1; VEGF: vascular endothelial growth factor; SD: standard deviation; * *p* < 0.05, ** *p* < 0.01.

**Table 5:** Multiple regression analysis showing independent associations between monocyte markers expression and metabolic parameters or inflammatory status.

Metabolic Parameters	CD14dimCD16++			CD14+CD206+CD163+		
	Standardized Coefficient β	95% Confidence Interval	*p*-Value	Standardized Coefficient β	95% Confidence Interval	*p*-Value
**BMI (kg/m^2^)**	0.19	−0.27 to 0.52	0.51	4.02	−67.74 to 34.37	0.15
**BP/diastolic (mmHg)**	0.20	−0.43 to 0.41	0.96	6.30	−58.51 to 101.6	0.18
**Resting Heart Rate (bpm)**	0.11	0.03 to 0.50	0.03 *	7.59	−91.12 to 101.7	0.61
**Triglycerides (mmol/l)**	3.07	−6.41 to 6.40	0.99	479.50	−6525 to 5661	0.53
**Total cholesterol (mmol/l)**	1.16	−0.90 to 3.94	0.21	144.70	−1706 to 1971	0.53
**HOMA-IR**	4.02	0.64 to 17.42	0.04 *	157.40	−2001 to 1998	0.99
**C-Peptide (pg/mL)**	3.45	2.14 to 16.54	0.01 *	290.30	−3584 to 3793	0.78

BMI: body mass index; BP: blood pressure; HOMA-IR: homeostatic model assessment of insulin resistance; C-peptide: connecting peptide; SD: standard deviation; * *p* < 0.05.

## Supplementary Materials

The following are available online at https://www.mdpi.com/2073-4409/9/5/1189/s1, Figure S1: Representative figure for FACS analysis, Table S1: Descriptive characteristics of the study population stratified by sex.
