# Supplementary material for: Increasing the Duration of Light Physical Activity Ameliorates Insulin Resistance Syndrome in Metabolically Healthy Obese Adults
Source: Cells. 2020 May 11;9(5):1189. doi: 10.3390/cells9051189 (PMC7290973; doi:10.3390/cells9051189)
Supplement: Supplementary file 1 [file cells-09-01189-s001.pdf]

Monocyte subset gating strategy

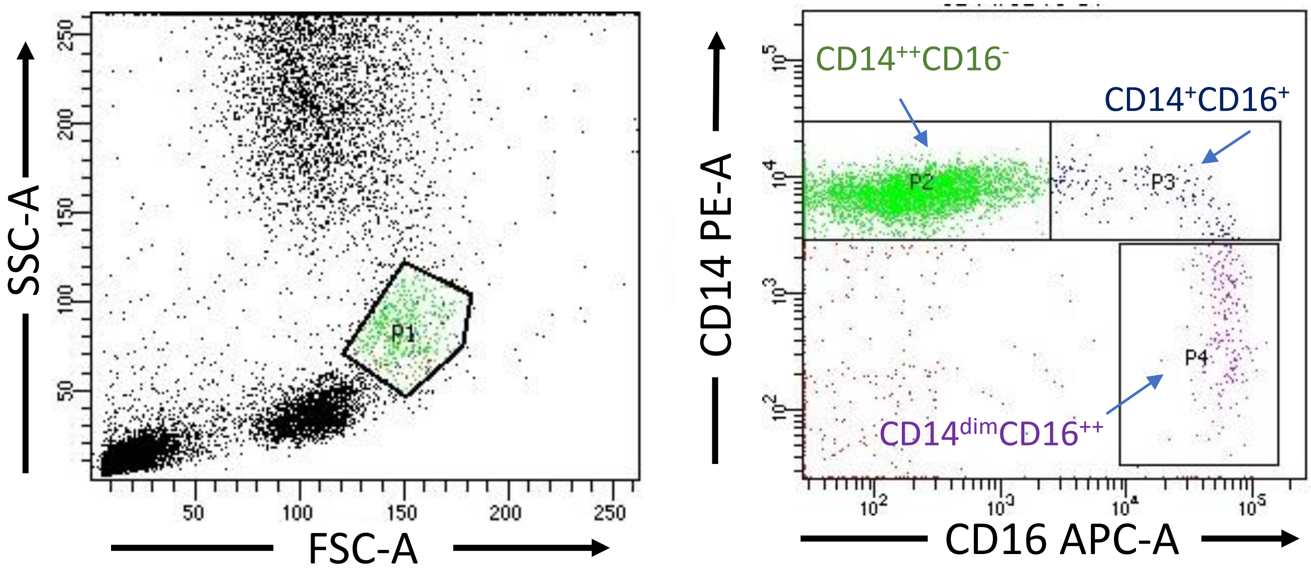

Supplementary Figure S1. **Monocyte subset gating strategy**. Gating monocyte subsets according to their size and granulation, CD14 and CD16 expression was used to identify the three monocyte subsets including the (P2) classical subset (CD14++CD16−), the (P3) intermediate subset (CD14++CD16+), and the (P4) non-classical subset (CD14dimCD16++).

Table S1 Descriptive characteristics of the study population stratified by sex

Physical characteristics of subjects	Male	Female	P-value
	(N= 30)	(N=30)	
**Age (years)**	33.6 ± 5.65	33.00 ± 4.08	0.8213
**Weight (kg)**	102.6 ± 9.9	88 ± 9.7	**0.0205***
**Height (cm)**	174.15 ±8.73	162 ± 8.47	**0.0290***
**BMI (kg/m2)**	33.16 ± 2.81	34 ± 3.15	0.8217
**Waist circumference (inch)**	45.78 ± 5.09	39 ± 4.06	**0.0102***
**Hip circumference (inch)**	46.94 ± 3.39	47 ± 3.56	0.9346
**Fat weight (kg)**	34.05 ± 8.14	38 ± 7.05	0.7783
**lean weight (kg)**	61.14 ± 7.59	50 ± 7.58	**0.0063****
**fat %**	31.16 ± 4.42	39 ± 3.8	**0.0142***
**BP/ systolic (mmHg)**	123.9± 10.84	115 ± 7.4	0.1077
**BP/diastolic (mmHg)**	75 ± 8.9	73 ± 9.96	0.6958
**HR**	76.4 ± 8.44	77 ± .16	0.8805
**Fasting concentration of serum samples (n=60)**	Male	Female	P-value
	(N= 30)	(N=30)	
**Fasting glucose (mmol/l)**	5.37 ± 0.726	5.0 ± 0.731	0.943
**Triglycerides (mmol/l)**	0.93± 0.22	1.0 ± 0.25	0.526
**Total cholesterol (mmol/l)**	5.47 ± 1.07	5.0 ± 0.731	0.5821
**HDL cholesterol (mmol/l)**	1.31 ± 0.240	1.0 ± 0.257	0.795
**Insulin Con. (mu/l)**	2.46 ± 1.54	4 ± 1.19	0.121
**HOMA-IR**	0.9 ± 0.40	1 ± 0.043	0.895
**C-Peptide (pg/ml)**	1.40 ± 0.502	2 ± 0.46	0.434

All values are means + standard deviations unless labeled otherwise
